# A thermosensitive electromechanical model for detecting biological particles

**DOI:** 10.1038/s41598-019-48177-2

**Published:** 2019-08-12

**Authors:** Masoud SoltanRezaee, Mahdi Bodaghi, Amin Farrokhabadi

**Affiliations:** 1 Young Researchers and Elites Club, Science and Research Branch, Islamic Azad University, Tehran, Iran; 2 Department of Engineering, School of Science and Technology, Nottingham Trent University, Nottingham, NG11 8NS United Kingdom; 3 Department of Mechanical Engineering, Tarbiat Modares University, Tehran, Iran

**Keywords:** Mechanical engineering, NEMS

## Abstract

Miniature electromechanical systems form a class of bioMEMS that can provide appropriate sensitivity. In this research, a thermo-electro-mechanical model is presented to detect biological particles in the microscale. Identification in the model is based on analyzing pull-in instability parameters and frequency shifts. Here, governing equations are derived via the extended Hamilton’s principle. The coupled effects of system parameters such as surface layer energy, electric field correction, and material properties are incorporated in this thermosensitive model. Afterward, the accuracy of the present model and obtained results are validated with experimental, analytical, and numerical data for several cases. Performing a parametric study reveals that mechanical properties of biosensors can significantly affect the detection sensitivity of actuated ultra-small detectors and should be taken into account. Furthermore, it is shown that the number or dimension of deposited particles on the sensing zone can be estimated by investigating the changes in the threshold voltage, electrode deflection, and frequency shifts. The present analysis is likely to provide pertinent guidelines to design thermal switches and miniature detectors with the desired performance. The developed biosensor is more appropriate to detect and characterize viruses in samples with different temperatures.

## Introduction

In recent decades, deformable electrodes have been considered as essential parts of several electromechanical systems, biological sensors, smart structures, and thermal switches^1–5^. These instruments in the ultra-small scale have a wide range of applications, which changes from microscale detection of forces to mass detection of molecules^6–8^. Most of such devices utilize beam-based structures to recognize signals with wide magnitudes range, measure the cell weight, and determine the operational ranges. On the other hand, scientists have made serious attempts to examine the properties of different biological particles, including molecules, biocells, viruses, or bacteria via these tiny instruments, which have opened an important area in the biomedical sciences^9,10^. With the high demand for ultra-sensitive biodetectors, beam-based micro and nanosystems have emerged and developed recently^11–15^. Their fabrication has also attracted much attention due to its difficulties in the ultra-small scale to reach acceptable accuracy in the instrumentation engineering^2,16^. Recently, it has been reported that fabricating nanobridges from the silicon crystal walls of 30 nm thickness is possible by the application of focused ion beam (FIB) and scanning electron microscopy (SEM) based techniques^17^.

New experimental advances in the ultra-small technology show the wonderful possibility of discovering, identifying and manipulating tiny particles within microsystems^18–20^. There are different techniques and devices such as optical tweezers^21^, magnetic tweezers^22^, surface plasmon resonators^23^, and thermo-electro-mechanical sensors^24^ for manipulating and examining the properties of cells and biological molecules. Each scientific instrument has unique applications and advantages; however, there is no comprehensive device. Thermo-electro-mechanical biosystems are thermosensitive devices that do not need any laser beam, magnetic field, light source/detector, or special equipment. In this field, micro and nanoelectromechanical systems (MEMS and NEMS) now play significant roles because these structures can also detect biological particles and diagnose diseases as biosensors or smart systems^25–28^. For instance, piezoelectric microresonators have been used in several hearing devices due to their high sensing performance^29–31^.

Generally, various significant building blocks in M/NEMS consist of at least two conductors, a substrate plate and a deformable arm. The electrostatically actuated deformable arm gradually deflects toward the fixed plate as the applied voltage increases. When the external voltage increases beyond a critical value, the electrical attraction becomes larger than the corresponding restoring forces, which leads to the collapse condition. In the phenomenon known as the pull-in instability, the critical voltage is called the pull-in voltage. Studying this phenomenon, which can restrict the operational range, and determining its characteristics are essential in the modeling and analysis of micro and nanosystems.

For design purposes of miniature switches and detectors, many trials are required to obtain an anticipated quality and performance such as dimensions of structures and the nature of internal material microstructures^30^. The emergence of extra impacts through the change of the scale order will also cause a number of additional important issues in the ultra-small scale. It has been demonstrated that we can achieve a better quality factor and sensitivity performance by decreasing the size of system^10,32^. Therefore, the size dependency of internal material at small scale is a main key that should be reflected in the simulation of miniature systems accurately. Moreover, the surface layer energy can affect the resonators and become more dominant in micro and nanoscales^33,34^. Owing to the considerable ratio of the outer surface area of a miniature detector to its volume, this parameter can make a significant contribution to the structural response. It has recently been demonstrated that by minimizing the surface stress we are able to further improve the mass sensitivity of clamped-clamped microresonators^35^. In addition, the effect of thermal stress on the pull-in instability and the frequency of clamped microbeams/plates have been less considered; however, it significantly affects their behaviors^36,37^.

The miniature systems play a major role in the biological and measurement applications due to their outstanding electrical, mechanical, and thermal characteristics^38^. Aboelkassem *et al*.^1^ modeled the behavior of a biomass sensor by considering a single cell mass via a microbeam. The operating principle was based on detecting the shift in the system natural frequency^18^ to measure the mass of a cell deposited on the sensing tip. Recently, an actuator has been examined for mass detection of biocells and nanocrystalline materials characterization by Shaat and Abdelkefi^10^. For disease diagnosis aim, a MEMS actuator was suggested to detect the human immune-viruses (HIVs) and determine the existing number of viruses in a blood sample. Later, they^39^ developed a nanocrystalline silicon antibody-coated cantilever to investigate the pull-instability and sensitivity of biocell systems by using modified couple stress theory (MCST). The results of this review indicate the importance of pull-in instability and frequency analysis on the investigation of biosystems behavior.

Generally, a layer or an array of biological particles can be captured by the coated materials on the surface of both fixed and deformable components of micro and nanosystems^40,41^. Two well-known conditions by considering the deposited particles exist in the beam-based devices. First, a biological entity is deposited on the tip or middle of M/NEMS movable conductor, which has been studied in detail extensively^1,10,35,38,39,42,43^. Second, an entity (an array of cells) or any other matter is deposited on the substrate that can block the electrostatically actuated length of the ground conductor. In this case, the actuated sensing zone has been limited. This phenomenon has less been investigated in miniature resonators, which affects the quality factor, pull-in parameters, and system frequencies of biodetectors significantly. For example, it was demonstrated that the blocked zone might prevent the pull-in instability in cantilever switches^44^.

In this paper, we present a model with capability of estimating thermo-electro-mechanical behaviors of MEMS biodetectors. The number of particular particles or the size of an unspecified cell can be obtained by investigating the threshold voltage, electrode deflection, and frequency shift. Here, the simultaneous contributions of surface layer energy, material size-dependent, and thermal expansion are considered to develop an accurate model and achieve a valid parametric study. It demonstrates that the coupled effects of all above-mentioned issues on the performance and sensitivity of a thermo-electro-mechanical biodevices are essential, which have not simultaneously been analyzed yet. The static and dynamic responses of the system model are validated by available experimental, analytical, and numerical results. Finally, a frequency analysis is carried out to detect biological entities especially viruses by evaluating the frequency shift of the system at different sample temperatures. The obtained results are expected to be instrumental in instability analysis, functional design and applications of numerous biocell sensors, band-pass filters, and thermal gates/switches.

## Model

**Figure 1:**
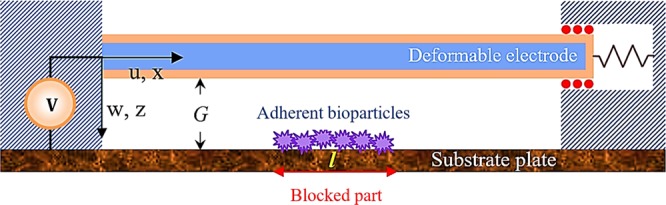
Schematic of the proposed biodetector subjected to the deposited array of biological particles on the coated sensing zone.

Figure 1 shows the schematic of a doubly supported (DS) beam fabricated from a movable electrode and a fixed plate as a substrate. The system has been powered by an external DC power supply to increase its sensing performance. In the proposed system, the length of the blocked section due to the deposited biological particles is *l*. Moreover, the width, thickness, and length of the beam are *b*, *h*, and *L*, respectively. Note that boundary conditions (BCs) of the DS structure are such that the beam does not tolerate any traction along its neutral axis. It means that the beam’s left end is clamped; however, the right end can just move horizontally without any rotation. In addition, a spring is connected to the sliding end as another control parameter to increase the adjustability of the system. It should be noted that, as fully clamped electrode is stiff, its sensitivity is not suitable to be used as a biosensor. On the other hand, thermal stress is meaningless without considering the spring in such a configuration. Such a configuration is applicable in adjustable microgates, tunable filters, thermal switches, and mass sensors^34,35,45,46^. Furthermore, the substrate plate is coated with a particular antigen material that is used as an attractor to detect biological particles^40^. For the sake of simplicity, it is assumed that the array of particles is deposited in the middle of the fixed conductor symmetrically^35^. It should be noted that in most of practical cases, biological sensors are used out of the live bodies. Usually a small sample is taken from the body and tested by biosensors. Regarding our developed sensor, particles cannot be kept alive after test. In this research, we will focus on introducing a thermosensitive beam-based microelectromechanical device with the ability of detecting viruses. In this model, the biological particles are adhering on the fixed substrate and detecting is implemented by measuring and investigating the system frequency shift, threshold voltage, and electrode deflection using the non-classical beam theory.

### Strain, potential, and kinetic energy

The strain energy of a thermal switch by considering the temperature variation, as stated in the general theory of thermal elasticity mechanics, can be written as^47^.

1 $${U}_{t}=-\frac{EA\,\Delta T}{2}{\alpha }_{t}{\int }_{0}^{L}{(\frac{\partial w(x,t)}{\partial x})}^{2}dx,$$

where *E* and *A* are Young’s modulus and rectangular cross-sectional area of the electrode (see Fig. 1). The terms Δ*T* and *α*_*t*_ denote the temperature variation and thermal expansion coefficient, respectively.

When an electrostatic attraction applies across the conductive plate and deformable electrode, the arm will endure distortion. Afterward, the geometrical nonlinear deformation will happen possibly. The axial strain of the electrode *ε*_0_ at its neutral axis is given by^48^

2 $${\varepsilon }_{0}(x,t)=\frac{ds-dx}{dx}={({(1+\frac{\partial u(x,t)}{\partial x})}^{2}+{(\frac{\partial w(x,t)}{\partial x})}^{2})}^{0.5}-1.$$

where *s* is the actual length of arched beam during deflection.

It can be stated here that the potential energy of the linear spring is obtained as

3 $${U}_{k}=\frac{1}{2}{K}_{s}{u}^{2}(x,t),$$

where *K*_s_ is the spring stiffness.

The nonlinear curvature *ζ* is given by (*θ* is angle of an element of the arched beam)^48^

4 $$\zeta (x,t)=\frac{d\theta }{ds}.$$

Note that for beam-based resonators with one end hinged or fixed and the other end sliding or free, the strain across the natural axis will be equal to zero^48^. Having Eq. (4) and applying the relation *ε*_0_ = 0, expanding the result by employing Taylor series and removing the higher-order terms. It is convenience to substitute the nonlinear curvature with a relation of the beam transverse displacement. Hence, the nonlinear curvature will be derived as

5 $$\zeta (x,t)=\frac{{\partial }^{2}w(x,t)}{\partial {x}^{2}}+\frac{1}{2}{(\frac{\partial w(x,t)}{\partial x})}^{2}\frac{{\partial }^{2}w(x,t)}{\partial {x}^{2}}.$$

By taking the nonlinear curvature of such systems, both of axial strain ***ε*** and stress *σ* tensors cab be defined as^49,50^

6 $${\varepsilon }_{xx}(x,t)={\varepsilon }_{0}(x,t)-z\zeta (x,t);\,\,{\rm{all}}\,{\rm{other}}\,{\varepsilon }_{ij}=0,$$

7 $${\sigma }_{xx}(x,t)=E{\varepsilon }_{xx}(x,t);\,\,{\rm{all}}\,{\rm{other}}\,{\sigma }_{ij}=0.$$

On the other hand, the stress in the surface area of the microbeam can be stated as^50,51^

8 $$\tau (x,t)={\tau }_{0}(x,t)+{E}^{s}{{\varepsilon }_{xx}}^{s}(x,t),$$

where *τ*_0_ is the residual stress of the surface, *E*^*s*^ and *ε*_*xx*_^*s*^ are the surface Young’s module and strain, respectively.

Applying the mentioned stresses, the bending moment of the microdetector will be derived as^50,51^

9 $$M={\int }_{A}{\sigma }_{xx}zdz+{\int }_{S}({\tau }_{0}+{E}^{s}{{\varepsilon }_{xx}}^{s})zdz=-\zeta (x,t)(E{I}_{b}+{E}^{s}{I}_{s}),$$

where *I*_*b*_ and *I*_*s*_ are related to the second moment of area of the bulk and surface layer of the electrode, respectively.

As another result, the mechanical strain energy of beams, including the surface layer terms will be given by^51^

10 $${U}_{m}=\frac{{E}_{eff}{I}_{eff}}{2}{\int }_{0}^{L}{\zeta }^{2}(x,t)dx,$$

where

11 $${E}_{eff}{I}_{eff}=E{I}_{b}+{E}^{s}{I}_{s};\,{I}_{b}=\frac{b{h}^{3}}{12};\,{I}_{s}=\frac{b{h}^{2}}{2}+\frac{{h}^{3}}{6}.$$

When the curvature is nonzero, because of the residual tension in the surface, the associated strain energy arising from this load can be developed as

12 $${U}_{s}={\tau }_{0}b{\int }_{0}^{L}\zeta (x,t)\,w(x,t)dx.$$

Generally, in the submicron-scale, the classical theories of continuum mechanics will not be able to clarify the size phenomenon of ultra-small systems and structures. Consequently, new comprehensive theories have been developed and modified, e.g. MCST^52^. Based on the MCST, the strain energy resulting from the small scale effect can be stated as^53^

13 $${U}_{l}=\frac{1}{2}{\int }_{\Xi }{m}_{ij}{\kappa }_{ij}d\Xi ,$$

where $\Xi$ is an occupying zone of isotropic elastic material. Furthermore, *κ*_*ij*_ is the symmetric curvature and *m*_*ij*_ is the couple stress tensor.

Eventually, the strain energy resulting from the effect of material size is derived as^46^

14 $${U}_{l}=\frac{bhE{\ell }^{2}}{4(1+\nu )}{\int }_{0}^{L}{(\frac{{\partial }^{2}w(x,t)}{{\partial }^{2}x})}^{2}dx,$$

where *v* and *ℓ* are Poisson’s ratio and the internal material size-dependent parameter, respectively.

In addition, the kinetic energy of the system is (*ρ* is the beam density)

15 $${E}_{k}=\frac{1}{2}{\int }_{0}^{L}\rho A{(\frac{\partial w(x,t)}{\partial t})}^{2}dx.$$

### External work

Generally, the performed work by all the distributed external force (*F*_*ext*_) can be stated as

16 $${W}_{ext}={\int }_{0}^{L}{F}_{ext}w(x,t)\,dx.$$

Taking into account the fringing correction for the electric field, the electrostatic attraction can be given by^54^

17 $${F}_{els}=\frac{0.65{\varepsilon }_{0}\,{V}^{2}}{2(G-w(x,t))}+\frac{{\varepsilon }_{0}b{V}^{2}}{2{(G-w(x,t))}^{2}}$$

where *ε*_0_ = 8.854e-12 F.m^−1^ is the permittivity factor of free space. It has been checked that dispersion forces do not affect significantly on the system behavior.

As mentioned, living cells, antibodies, viruses, or bacteria can deposit on the sensing zone of the substrate plate due to the coated material that used as an attractor to detect particles. In this case, we will face piecewise external load in the present microswitch. As a result, the deposited particles affect the sensitivity of biodetectors significantly, which must be reflected into the modeling. By assuming the symmetric deposition of particles in the middle of the fixed electrode, the electrostatically actuated length will be controlled by the Heaviside function as

18 $$H(x)=1-H(x-(L-l)/2)+H(x-(L+l)/2),$$

where *l* is the length of the middle symmetrical blocked piece of the substrate.

Since the studied resonator is under external electrostatic attractions, the performed work by these forces is given by

19 $${W}_{ext}={\int }_{0}^{L}({\int }_{0}^{w}{F}_{els}H(x)dw(x,t))dx.$$

### Governing equations

In order to establish the motion equations (EOM), the extended Hamilton’s principle can be used as

20 $$\delta {\int }_{0}^{t}({U}_{t}+{U}_{k}+{U}_{m}+{U}_{s}+{U}_{l}-{E}_{k}-{W}_{ext})dt=0.$$

By replacing the strain and kinetic energies as well as the work performed by electrostatic force into Eq. (20), the following equation can be derived

21 $$\begin{array}{l}(E\frac{b{h}^{3}}{12}+{E}_{s}(\frac{b{h}^{2}}{2}+\frac{{h}^{3}}{6}))[\frac{{\partial }^{4}w}{\partial {x}^{4}}+\frac{\partial }{\partial x}(\frac{\partial w}{\partial x}\frac{\partial }{\partial x}(\frac{{\partial }^{2}w}{\partial {x}^{2}}\frac{\partial w}{\partial x}))]+\frac{bhE{\ell }^{2}}{2(1+\nu )}\frac{{\partial }^{4}w}{\partial {x}^{4}}\\ +\,(bhE\,\Delta T{\alpha }_{t}-{\rm{sign}}(\Delta T)\frac{{K}_{s}}{2}{\int }_{0}^{L}{(\frac{\partial w}{\partial x})}^{2}dx)\frac{{\partial }^{2}w}{\partial {x}^{2}}-b{\tau }_{0}\frac{{\partial }^{2}w}{\partial {x}^{2}}(2+{(\frac{\partial w}{\partial x})}^{2})+\rho bh\frac{{\partial }^{2}w}{\partial {t}^{2}}\\ =\,(\frac{0.65{\varepsilon }_{0}{V}^{2}}{2(G-w(x,t))}+\frac{b{\varepsilon }_{0}{V}^{2}}{2{(G-w(x,t))}^{2}})H(x).\end{array}$$

It is convenient to simplify the parametric calculations, so the governing equation is expressed in the nondimensional form by introducing the following terms

22 $$\begin{array}{l}\chi =\frac{x}{L},\,\varpi =\frac{w}{G},\,\tau =\frac{ht}{2{L}^{2}}\sqrt{\frac{E}{3\rho },\,}\xi =\frac{{G}^{2}}{{L}^{2}},\,\iota =\frac{6{\ell }^{2}}{(1+\nu ){h}^{2}},\,\lambda =\frac{24{\tau }_{0}{L}^{2}}{E{h}^{3}},\,\alpha =\frac{l}{L},\\ \vartheta =\frac{12\,{\rm{\Delta }}T}{{h}^{2}}{L}^{2}{\alpha }_{t},\,\kappa ={\rm{sign}}({\rm{\Delta }}T)\frac{6L{G}^{2}{K}_{s}}{Eb{h}^{3}}{\int }_{0}^{L}{(\frac{\partial \varpi }{\partial \chi })}^{2}d\chi ,\,\eta =\frac{2{E}^{s}}{E}(\frac{3}{h}+\frac{1}{b}),\\ \varphi =0.65\frac{G}{b},\,\upsilon =\frac{V{L}^{2}}{hG}\sqrt{\frac{6{\varepsilon }_{0}}{hGE}},\,\underline{H}=1-H(\chi -\frac{1-\alpha }{2})+H(\chi -\frac{1+\alpha }{2}).\end{array}$$

By replacing the dimensionless terms into Eq. (21), multiplying the results by *L*4**/**(*EIG*), the nonlinear equation can become nondimensional as

23 $$\begin{array}{c}(\frac{\varphi {\upsilon }^{2}}{1-\varpi }+\frac{{\upsilon }^{2}}{{(1-\varpi )}^{2}})\underline{H}(\chi )=(1+\eta +\iota )\frac{{\partial }^{4}\varpi }{\partial {\chi }^{4}}+(\vartheta -\kappa -2\lambda )\frac{{\partial }^{2}\varpi }{\partial {\chi }^{2}}\\ +\,\xi ((1+\eta )[\frac{\partial }{\partial \chi }(\frac{\partial \varpi }{\partial \chi }\frac{\partial }{\partial \chi }(\frac{{\partial }^{2}\varpi }{\partial {\chi }^{2}}\frac{\partial \varpi }{\partial \chi }))]-\frac{1}{2}\lambda {(\frac{\partial \varpi }{\partial \chi })}^{2}\frac{{\partial }^{2}\varpi }{\partial {\chi }^{2}})+\frac{{\partial }^{2}\varpi }{\partial {\tau }^{2}}.\end{array}$$

## Method

In the following, the Galerkin decomposition will be utilized to discretize Eq. (23) and obtain a suitable solution for the derived differential equation. Afterward, the discretization results should be solved numerically due to the inherence nonlinear behavior of the governing equation. In general, the nondimensional transverse displacement of the beam $\varpi$ will be defined as a linear combination of several modes. In these conditions, the approximate solution for the structure will be constructed as $\varpi =B{\boldsymbol{\phi }}(\chi )$, where *B* is the amplitude factor and

24 $${\phi }_{i}=(\sin ({\Upsilon }_{i}\chi )-\,\sinh ({\Upsilon }_{i}\chi ))\frac{cosh{\Upsilon }_{i}-\,\cos \,{\Upsilon }_{i}}{\sinh \,{\Upsilon }_{i}-\,\sin \,{\Upsilon }_{i}}+\,\cosh ({\Upsilon }_{i}\chi )-cos({\Upsilon }_{i}\chi ).$$

By considering Eqs (24) and (23), multiplying the whole equation by *φ*(*χ*) ultimately the intended equation can be derived as

25 $$n(B)=m\ddot{B}+((1+\eta +\iota ){K}_{1}+(\vartheta -\kappa -2\lambda ){K}_{3}+\xi (2(1+\eta ){K}_{2}-0.5\lambda {K}_{4}))B,$$

where *K*_i_ are related stiffness terms and the beam inertia and external excitation are

26 $$m={\int }_{0}^{1}{{\boldsymbol{\phi }}}^{2}d\chi ,$$

27 $$n(B)={\int }_{0}^{1}(\frac{\varphi {\upsilon }^{2}}{1-B{\boldsymbol{\phi }}}+\frac{{\upsilon }^{2}}{{(1-B{\boldsymbol{\phi }})}^{2}})\underline{H}(\chi ){\boldsymbol{\phi }}d\chi .$$

Here, the deflection of a doubly supported microelectrode because of an external voltage can be achieved by solving Eq. (28). Therefore, the EOM of the thermal microswitch by considering the piecewise electrostatic attraction due to deposited entities can be rewritten as

28 $$((1+\eta +\iota ){K}_{1}+(\vartheta -\kappa -2\lambda ){K}_{3}+2(1+\eta )\xi {K}_{2}-0.5\xi \lambda {K}_{4})B={n}_{els}\underline{H}(\chi ).$$

At the unstable conditions, the tangent stiffness will be singular (det(*K*) = 0). Therefore, we will have an appropriate way to calculate the pull-in instability parameters of detectors. For solving the differential equation of such miniature resonators, pull-in characteristics will be determined numerically.

Generally, the natural frequency of the miniature structures must be equal to zero, when the pull-in instability takes place. This process is a beneficial method to investigate the vibrating behavior of actuated system and finding the critical parameters, especially the threshold voltage and system frequency. Consequently, the frequency of the mechanical resonator can be achieved via the equation $\det \,(K-m{\omega }^{2})=0$.

Considering Eq. (25), the equal stiffness of the studied DS beam will be developed as

29 $$K=(1+\eta +\iota ){K}_{1}+6\xi (1+\eta ){K}_{2}+(\vartheta -\kappa -2\lambda ){K}_{3}-1.5\xi \lambda {K}_{4}-dn(B)/dB.$$

Accordingly, by considering the mentioned determinant and the dynamic governing Eq. (25) of the prepared MEMS with the ability of detecting bioparticles, the *i*-th step relation will be derived as

30 $${m}^{i}\ddot{B}+((1+\eta +\iota ){K}_{1}^{i}+(\vartheta -\kappa -2\lambda ){K}_{3}^{i}+2(1+\eta )\xi {K}_{2}^{i}-0.5\xi \lambda {K}_{4}^{i})B={n}_{els}^{i}\underline{H}(\chi ).$$

## Results and Discussion

After developing the model, a parametric study of the presented thermal microswitch with an aim of investigating the system behavior to detect biological particles correctly will be carried out. Hence, we analyze the impacts of nonactuated zone due to the deposited particles, temperature variations, and molecular effects, on the performance of biodetectors. Note that it has been comprehended that by considering three modes, the convergence of the acquired results is appropriate. Consequently, the obtained results will be based on three basic functions.

In order to validate the results experimentally, Table 1 compares the obtained results for the microbeam deflection versus the external voltage of a cantilever resonator with both experimental and analytical results^55^. The material properties and dimensions of the considered microsystem are reported as *G* = 92 μm, *b* = 5 mm, *h* = 57 μm, *L* = 20 mm, and *E* = 155.8 GPa^55^. It can be concluded that the acquired results are in a good agreement with available experimental ones.

**Table 1:** Comparison of the sensor deflection with available experimental and analytical results.

Applied voltage (volt)	20	40	60	65	Pull-in
Analytical^55^	90.2	84.3	71.5	67.2	66.8
Experiment^55^	90.5	84.6	70.0	64.0	68.5
Numerical (SSLM)	90.19	84.10	69.31	60.37	66.87
Error ratio (%)	0.3	0.6	1.0	5.7	2.3

In another case, we have compared the threshold voltages with the experimental^56^, analytical^47^, and numerical^57^ results reported in the literature for beams with different length (Table 2). The width and thickness of deformable electrodes are 50 μm and 1 μm, respectively, and the initial gap is 3 μm. Furthermore, the modulus of elasticity and Poison’s ratio are 169 GPa and 0.6, respectively. As it can be seen, the results obtained from our model show an excellent correlation with experiments validating its high accuracy.

**Table 2:** Comparison of the sensor threshold voltage with the experimental, analytical and numerical results from the literature.

Technique	Voltage (L = 250 μm)	Voltage (L = 350 μm)
Experiment^56^	39.5 V	20.20 V
Analytic^47^	39.4 V	20.10 V
Galerkin^57^	39.3 V	20.07 V
Present work	39.6 V	20.40 V

To further verify the model, the measured dynamic pull-in voltages for four different electrode lengths are reported in Table 3. The electrode width, thickness, and initial gap is 100 μm, 1.5 μm, and 1.18 μm, respectively. Furthermore, the modulus of elasticity and Poison’s ratio are 151 GPa and 0.3, respectively. The pull-in voltage was only measured on one location (i.e., manipulators length 210 μm, 310 μm, 410 μm, and 510 μm). We have compared the results with available experimental data^58^ and the error have been calculated. A good correlation between the results for the dynamic problem is observed.

**Table 3:** Comparison of the sensor voltage with experimental dynamic responses^58^.

Electrode length (μm)	210	310	410	510
Experimental dynamic critical voltage (V)	27.95	13.78	9.13	6.57
Simulation dynamic critical voltage (V)	27.35	14.28	9.35	6.90
Error ratio (%)	2.1	3.6	2.4	5.0

The detailed dimensions as well as constants used for simulation of the present miniature biodetector are reported in Table 4. Due to the dimensions of the considered MEMS device, it generally has the ability to detect and characterize bioparticles such as several viruses, which is the main purpose of this research work. In the following, all the geometrical and material parameters characteristics are fixed unless otherwise specified.

**Table 4:** Specifications of the microswitch **(a)** material properties^47^ **(b)** dimensions.

	Parameter	Young’s modulus	Poisson’s ratio	Size parameter	Surface stress	Surface elasticity	Coefficient of thermal exp.
	Symbol	*E*	*v*	*ℓ*	*τ*_0_	*E* ^S^	*α*_t_
**(a)**	Value	68.5 MPa	0.3	30 nm	0.90 N/m	6.09 N/m	−7.4 × 10^−6^ K^−1^
	**Parameter**	**Electrode width**	**Electrode thickness**	**Electrode length**	**Initial gap**	**Spring stiffness**	
	Symbol	*b*	*h*	*L*	*G*	*K*_S_	
**(b)**	Value	0.1 μm	0.1 μm	5 μm	0.3 μm	5 N**/**m	

### Stability analysis

The chief aim of using several biodetectors is to attract biological particles on the coated zone and estimate the number/dimension/location/mass of particular entities. The basic idea that motivates the use of electrostatically actuated systems is to inspect the change in mechanical system behaviors due to adherent entities and to extract their characteristics.

**Figure 2:**
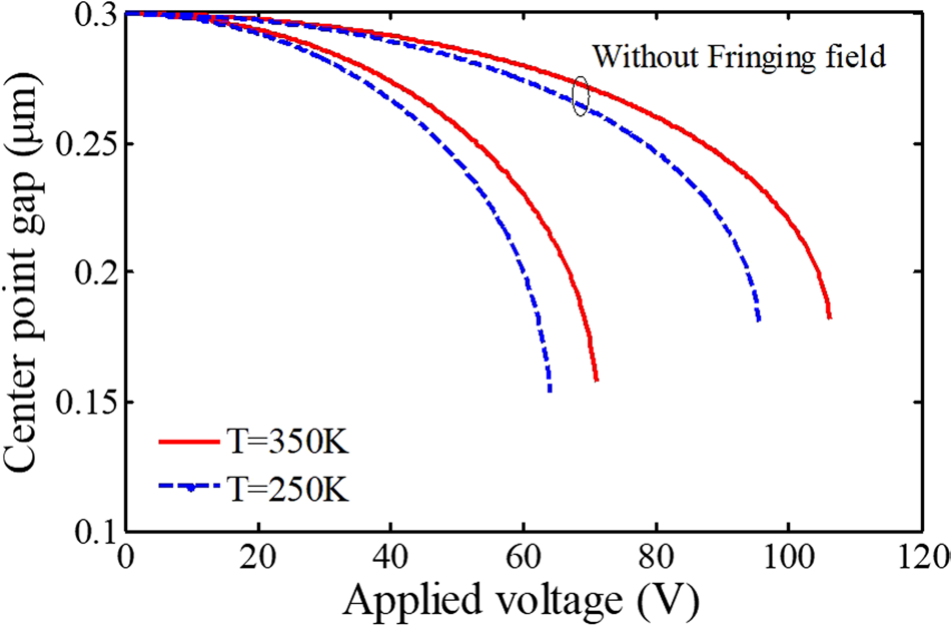
The impact of fringing correction on the sensor behavior at different temperatures.

The relationship between the center point displacement and the external voltage is revealed in Fig. 2 to analyze the sensing performance of thermal switches. According to the figure, the critical point deflection increases with increasing the applied voltage. Moreover, the graphs with and without consideration of the fringing field correction are plotted. It can be understood that neglecting this effect in the ultra-small scale can cause significantly incorrect results and the calculated critical voltage becomes overestimated. Figure 2 also demonstrates the effect of temperature rise on the instability parameters and detection sensitivity. It is seen that microsystems are greatly affected by the change in the environment temperature and the thermal load during practical applications. By increasing the temperature, the beam becomes more stable, so the critical voltage increases. It means that decreasing the temperature improves the sensitivity of thermal switches. In addition, without consideration of the fringing field, the effect of temperature variations will be more considerable; however, the pull-in instability deflection of the electrode will be reduced. These both are not appropriate for an ultra-sensitive detector that should take into account for a reliable design and accurate simulation.

**Figure 3:**
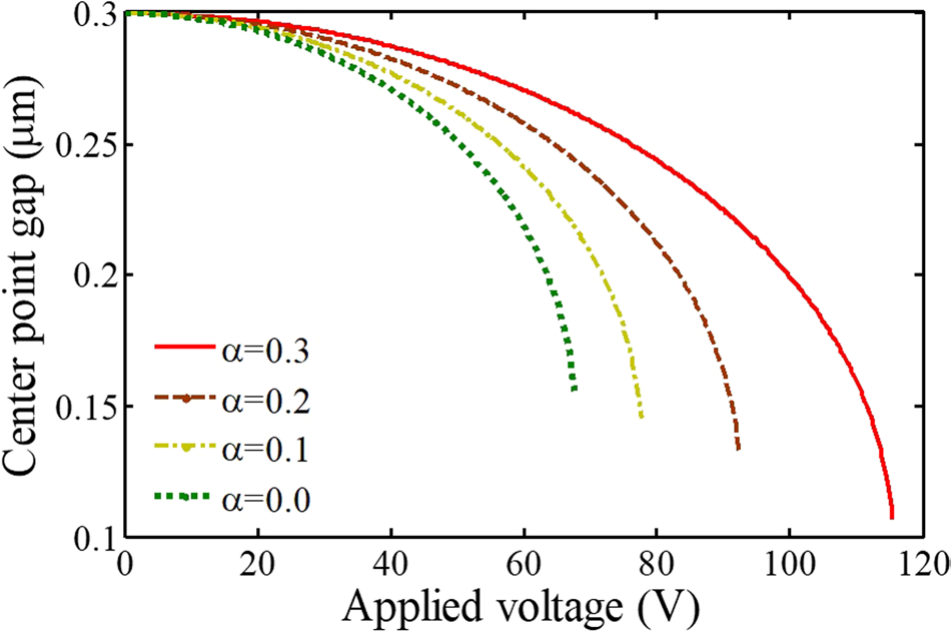
The impact of adherent biological particles on the sensor behavior (T = 300 K).

**Figure 4:**
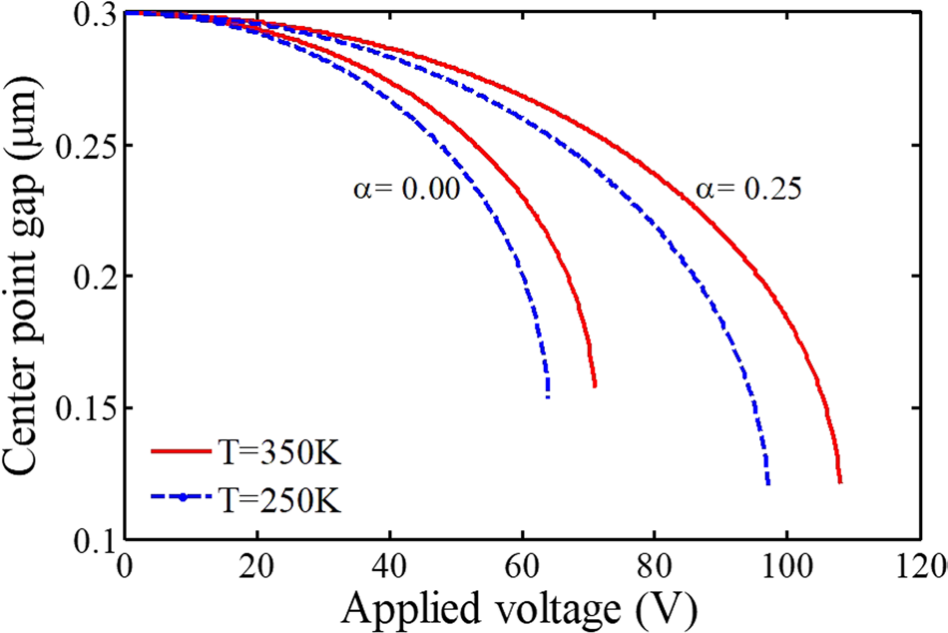
The impact of adherent biological particles on the sensor behavior at different temperatures.

Figures 3 and 4 include notable curves to show the effects of adherent biological particles on the sensing platform. By comparison of the considered curves, it is recognized that the length of the deposited entities, affect the instability parameters, sensing performance, and system behavior dramatically. In a case with an array of deposited particles, the effective electrostatically actuated length of the fixed electrode is shorter than the deformable electrode, i.e. the ratio of blocked actuated length *α* (=*l*/*L*) does not equal zero. As a result, its attraction is less than an electrode without deposited entities. In this situation, the system instability conditions take place with a delay owing to the blocked sensing zone of the substrate (Fig. 1). Here, both the pull-in voltage and deflection of the biosensor with such an electrode are larger than an unblocked electrode. It means the pull-in parameters increase with an increase in the blocked length of the substrate plate. Therefore, increasing the number of deposited particles or depositing larger particles leads to an increase in the threshold deflection and voltage of the electrode. This can be a significant point to identify the system behavior and obtain a valid parametric study.

Conversely, we can achieve an important idea to find out the dimension/number of deposited entities from a practical point of view. The dimension/number of adherent particles can be identified by analyzing the delay of the pull-in instability and evaluating both the threshold voltage and deflection. As mentioned, increasing the deposited particles results in an increase in the necessary voltage range to achieve the same deflection. Therefore, by estimating the pull-in voltage and/or the instability deflection, we will be able to predict the blocked length. Afterward, we can determine the size of the deposited particles or their number when we know particular bioparticles. Consequently, this will enable appropriate and easy detection and estimation of the number of particular cells or the dimension of unspecified entities, deposited on the device sensing zone.

In addition, the effect of temperature rise on the pull-in characteristics becomes more significant for a biodetector that has piecewise electrostatic attraction due to adherent particles. As a result, pull-in deflections as well as the difference between the pull-in voltages are enhanced remarkably by increasing the temperature. Finally, note that in biomass detectors to diagnose an attached particular disease on the deformable electrode, increasing the number of antibodies improves the sensitivity^38,39,42,43^; however, the deposited entities on the substrate decrease the sensitivity of biodetectors.

Investigating the effects of electrode dimensions on the detection sensitivity is important in biosensors. Examining the governing equations reveals that increasing the electrode cross-sectional area enhances its mechanical stiffness. Therefore, the pull-in voltage and system stability increase with the increase of the thickness and/or width, unlike the electrode length. Moreover, the sensitivity of electrostatically actuated systems will be increased by decreasing (increasing) the electrode width (length), which should be considered in the design of biological devices. Another point is that decreasing the thickness of biodetectors results in increasing the sensitivity of such devices, which has also been found in biomass cantilever sensors^10^. Finally, it should be mentioned that the effects of electrode width on the system behavior is more considerable than the electrode thickness especially due to accounting the fringing field correction.

### Frequency analysis

**Figure 5:**
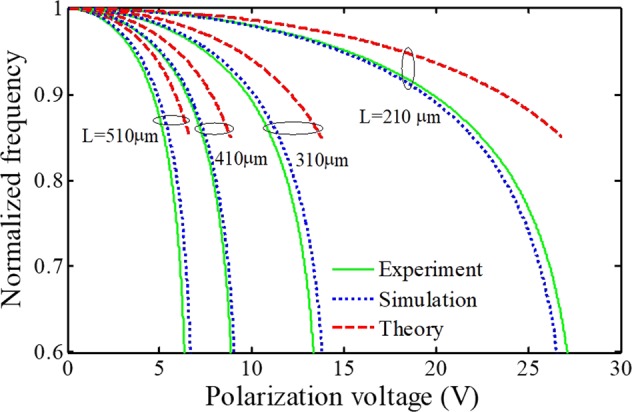
Frequency validation with available experimental and theoretical results^58^: normalized frequency vs. polarization voltage of manipulators with different electrode length.

In the following, detecting performance of thermal switches is investigated by evaluating the change in the magnitude of the dynamic responses. First, in order to verify the dynamic behavior of the present model, we validate the obtained results with available experimental and theoretical data for different cases (electrode length: 210, 310, 410, and 510 μm). The electrode width, thickness, and initial gap is 100 μm, 1.5 μm, and 1.18 μm, respectively. Furthermore, the modulus of elasticity and Poison’s ratio are 151 GPa and 0.3, respectively. In Fig. 5, the simulation results for frequency analysis of the present system model by considering electrodes with different length are compared with reported experimental and theoretical results. The better agreement between the present results than theoretical ones with experimental data confirms the accuracy of the present developed model.

To characterize the behavior of the prepared miniature biosensor, the normalized vibrating frequency Ω(=ω**/**ω_0_) of miniature biosensors related to the nondimensional voltage parameter *υ* is illustrated with the variations in each of the length-scale, and surface layer characteristics. Here, the results generally demonstrate that the frequency decreases as the electrostatic attraction increases and approaches zero as the instability happens. Consequently, the electrostatic DC attraction tends to soften the actuated system.

**Figure 6:**
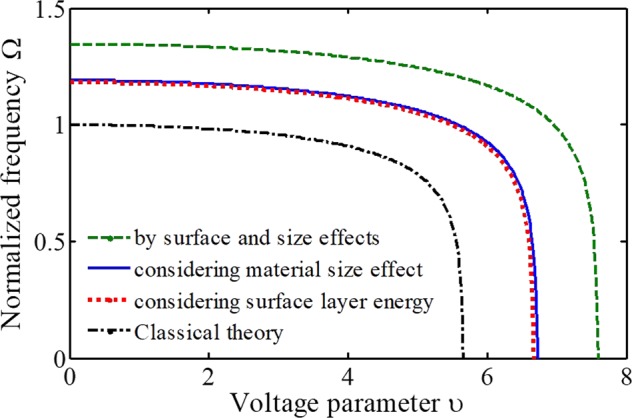
The impacts of surface layer energy and internal material size of the deformable electrode on the sensor behavior (T = 273 K).

It has recently been demonstrated that by minimizing the surface stress we are able to further improve the mass sensitivity of clamped-clamped microresonators^35^. However, system identification is required to get valid results in parametric study of bioMEMS. In order to model a micro and nanosystem perfectly, the classical model that has valid results in the macro-scale is unusable. Figure 6 displays the effects of surface layer energy and internal material size of deformable microelectrode on the frequency and dynamic response of the actuated biosensor. The obtained curves have been compared with the classical theory, which predicts the normalized frequency equals one. It can be found that without consideration of intermolecular effects in the micro and nanoscale, the system behavior cannot predict accurately, which is essential in ultra-sensitive manipulators. The results demonstrate the importance of taking simultaneous contributions of size and surface effects into account for an accurate simulation of mechanical biosensors using the non-classical theory. As a final point, using the relation $\lambda =24{\tau }_{0}{L}^{2}/(E{h}^{3})$, it can be understood that the effect of the residual surface parameter will increase by increasing the ratio of electrode length to thickness of the structure. Consequently, this effect is more noteworthy for biological detectors with a slender microelectrode.

**Figure 7:**
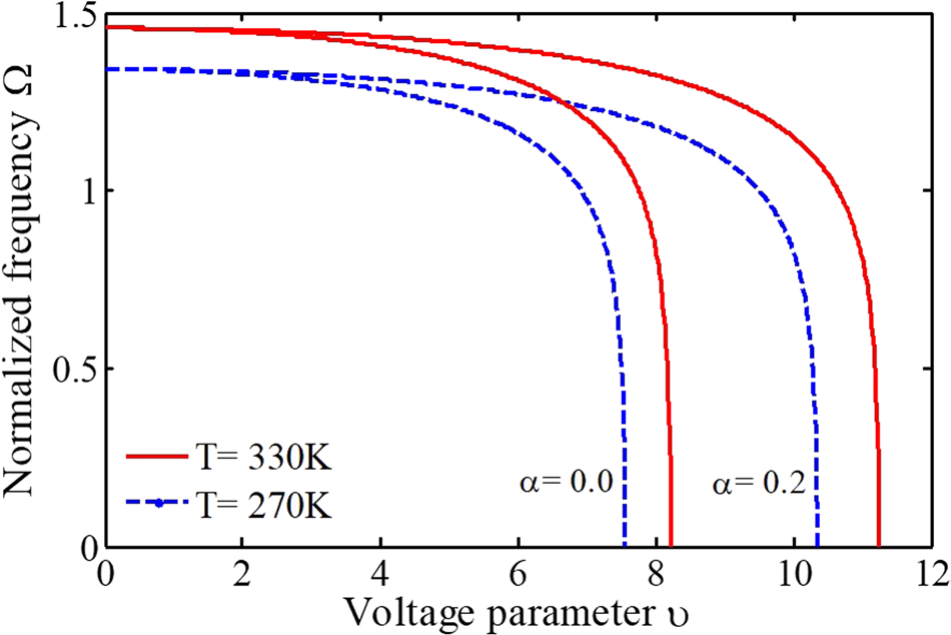
The impact of adherent biological particles on the sensor behavior at different temperatures.

By demonstrating the key roles of surface layer energy and internal material size on the sensing performance, we analyze the effects of adherent biological particles at different temperatures by considering small-scale effects in the following. The variation of the temperature on the stability and frequency of biosenosrs is observed in Fig. 7. It can be found that the sample or environment temperature significantly affects the sensing performance of bioMEMS. With increasing the system temperature, the microelectrode becomes stiffer, so the pull-in voltage and frequency will be increased. The reason is that the coefficient of thermal expansion for the considered material *α*_t_ is not positive (it shrinks when heated)^47^. Generally, the system frequency changes by considering different initial conditions for the environment temperature and blocked sensing part, which should be taken into account for a more accurate analysis. However, the adherence of the biological entities on the substrate conductor does not affect the unactuated frequency (υ = 0), as expected. Moreover, it is realized that the deposition of bioparticles affects the pull-in parameters more than the frequency. It is seen that the influence of the blocked sensing part on the frequency becomes more evident with increasing the external voltage. Furthermore, the influence of temperature rise on the pull-in characteristics is more significant for a biodetector with the piecewise electrostatic attraction. As a result, the difference between the pull-in voltages is enhanced by increasing the temperature. In addition, the effect of increasing the blocked length on the voltage parameter will not change linearly. This point evidences the significance of implementing a nonlinear analysis of such biosensors to achieve accurate results.

Totally, the shift in the frequency of mechanical biosensors is quite encouraging because it reveals that detecting the frequency shift can make an appropriate measure of the dimension/number of adherent particle(s) from a practical point of view.

## Conclusion

A new thermo-mechanical model was presented to detect biological particles and determine their dimension/number by examining instability parameters and response frequency. The number of particular entities or the dimension of an unspecified cell, which deposited on the substrate, can be obtained. This electrostatically actuated model has the ability to characterize bioparticles by investigating the threshold voltage and electrode deflection. A part of the substrate that attracts biological entities such as viruses is considered as a blocked sensing zone when the bioparticles adhere on it. The accuracy of the model and solution method has been validated experimentally, analytically, and numerically in several cases.

It is found that increasing the critical voltage of MEMS detectors results from depositing biological particles, which displays excellent potential for biosensing. Increasing in the threshold deflection is another important point to detect and measure deposited entities. Bioparticles characteristics can be estimated at different sample temperatures by employing the change in the threshold voltage and deflection via this thermosensitive model. It is concluded that the performance and sensitivity of thermo-electro-mechanical biosensors extremely depend on their mechanical properties. To predict their behavior perfectly, correct simulation of the complicated biosystems by considering system parameters simultaneously is necessary.
